# Impacts of Climate Change on Human Health: Emerging Evidence and Call to Action

**DOI:** 10.21315/mjms2022.29.5.1

**Published:** 2022-10-28

**Authors:** Jemilah Mahmood, Renzo R Guinto

**Affiliations:** Sunway Centre for Planetary Health, Sunway University, Selangor, Malaysia

## Introduction

It is already widely known that the climate crisis is an existential threat that is already impacting every aspect of human society and the planet. Hence, action from all sectors is not only necessary but urgent. One area that requires immediate attention is the impact of climate change on human health. For the past three decades, there have been numerous efforts from the academic and policy worlds to highlight the immutable connection between human health and climate change. However, it was only during the 26th Conference of Parties (COP26) of the United Nations Framework Convention on Climate Change, which was held in November 2021 in Glasgow, Scotland, that health started to receive an appropriate level of attention, in large part due to the still-ongoing Coronavirus disease (COVID-19) pandemic. Under the leadership of the World Health Organization (WHO) a two-week series of activities and discussions was held, bringing together health professionals, researchers, policymakers and advocates to highlight the importance of health in the climate discourse. The COP26 health programme culminated with more than 50 countries making a commitment to build climate-smart national health systems that are both resilient to the growing impacts of climate change and sustainable, so that they do no further harm to the planet through carbon emissions and other types of pollution (1). We hope that Malaysia will join in this global campaign.

Climate change affects human health in many different ways. For example, extreme weather events such as typhoons and intense flooding result in water-borne diarrheal disease outbreaks or worse forced displacement of entire communities to new locations without reliable access to food, water and other life-sustaining services. Changes in environmental conditions such as precipitation and temperature influence the behaviour of disease-carrying mosquitoes, resulting in more outbreaks and reduced crop yields leading to hunger and malnutrition. Because of the diverse ways in which climate change influences health the magnitude of its health impact is challenging to measure or estimate. For instance, the WHO previously estimated that, due to climate change alone, there will be 250,000 additional deaths annually between 2030 and 2050 resulting from climate-sensitive diseases such as undernutrition, infectious diseases and heat-related illness (2). A newer study projected that if business as usual continues, an additional 83 million people will die from exposure to extreme heat alone by the end of the century (3). One certainty is that the direction of change in negative health outcomes due to climate change is upwards—more diseases, disabilities and even deaths in the years and decades to come.

Below, we summarise some of the latest research and events that must sound the alarm bells for us in the medical sector—emerging health challenges linked to climate change that we must pay attention to and urgently act on.

### Future Pandemics

The COVID-19 pandemic may not be over yet but we must already be alert to the possibility of future ones. Pandemics originate from zoonotic spillover events—when pathogens jump from animals to human beings. This means that we must tackle the major factors that tighten the animal-human interface where such spillover events do occur. One of the known drivers of infectious disease emergence is rapid and widespread urbanisation, which impinges and disturbs natural habitats where pathogen-carrying animals live. Other human activities that bring people closer to animals include wildlife consumption and trade as well as animal farming for meat production.

An added driver to all these human activities is the evolving climate crisis (which, let us remember, is also driven by human behaviour), which alters environmental conditions not only for humans and animals but also for pathogens with pandemic potential. Using machine learning, a recent study revealed that by 2070, there will be at least 15,000 cross-species transmission events of at least one novel virus that could occur between mammals, including human beings, if climate change continues to worsen and if the goals of the Paris Agreement are not achieved (4). The majority of these zoonotic spillovers will occur in Southeast Asia, which is a major biodiversity hotspot. Therefore, preventing the next pandemic in our own backyard must be a priority for Malaysia and the Association of Southeast Asian Nations (ASEAN). Currently, the WHO is negotiating a future pandemic treaty to ensure that COVID-19 does not happen again. This new treaty must, therefore, emphasise pandemic prevention through tackling the aforementioned infectious disease drivers, including climate change.

### Exposure to Extreme Heat

Over the past months, we have witnessed heat wave events wreak havoc in China, Central Asia and Europe, resulting to spikes in emergency room visits and even deaths. Being in a tropical region, we in Malaysia may be feeling complacent about the potential impacts of extreme heat on our health. One analysis estimates that under one of the climate scenarios, by 2050, between 600 million and 1 billion people in Asia will be living in areas that are likely to experience lethal heat waves each year (5). In major cities in other regions such as Athens and Los Angeles, local governments are already appointing ‘Chief Heat Officers’ that will lead the development of city health plans to increase community resilience to extreme heat—a policy innovation that we can easily emulate (6).

Moreover, extreme heat not only affects people directly but also other sectors on which human survival depends. For instance, the agriculture sector is also very sensitive to climate crisis, particularly to drought which can destroy croplands and jeopardise food security. Thus, there is an increasingly urgent need to enhance the resilience of the agricultural sector to climate change impacts—while also lowering its carbon emissions and ecological footprint. One of the proposed solutions is the adoption of the Planetary Health Diet—a diet that provides nutrition for all people while also protecting the planet (7). We must investigate the feasibility of this diet in Malaysia, given our unique cultural, geographic and social context.

### Climate Change and Mental Health

The physical health impacts of climate change, such as the diseases mentioned above, are much more widely known to the medical community and the broader public. What receives little attention is the impact of climate change on people’s mental and emotional wellbeing. In the disaster response and humanitarian sectors, there is some awareness of the importance of addressing post-traumatic stress experienced by victims of typhoons and other calamities. However, other indirect and subtle pathways by which climate change affects mental health are less well understood.

For instance, the concept of ‘climate anxiety,’ which includes a wide range of emotions about climate change, remains to be investigated especially in non-Western contexts (8). Particularly, vulnerable to climate anxiety are young people who are feeling increasingly concerned about their futures in a scenario of an unstable climate. One recent cross-sectional study of young people from 10 countries, revealed that the young people from the Philippines are the most climate-anxious in the world, possibly due to their direct experience of extreme weather events (9). This growing mental health pandemic driven by the climate crisis will impose an enormous additional burden on existing mental healthcare systems which are largely underdeveloped, deeply stigmatised and poorly resourced. This emerging area calls for more transdisciplinary collaborative research between psychologists, psychiatrists, social scientists and experts from other related disciplines—not only to assess the magnitude of this problem but also capture people’s lived experiences and develop interventions and policies that will enhance people’s mental resilience to the climate crisis.

## Conclusion

With these emerging health threats driven by climate change as well as existing health problems that the climate crisis will exacerbate, we, in the medical community must embrace climate change as our own issue of concern as well as part of our healing enterprise. The Malaysian medical community can begin by coming together to declare that while we talk about climate change, we are now faced with the reality of a climate crisis which constitutes a public health emergency. This will ensure that building a national climate-resilient health system becomes an integral part of our country’s health reform agenda.

Such a declaration can also be used for our advocacy activities with other sectors and disciplines, especially those that require urgent climate mitigation and adaptation actions such as the energy and food sectors. The above-mentioned climate-related health issues must also be incorporated into the teaching of medical and other health students, as they will be the ones who will be preventing and treating these diseases in the decades to come. Finally, our medical institutes and research funders must invest in climate and health research in order to study the unknowns about the climate-health nexus and develop evidence-based solutions that will protect our citizens from the health impacts of the climate crisis.
